# Antibacterial and Antibiofilm Activity of Closantel Against *Staphylococcus epidermidis*

**DOI:** 10.1002/mbo3.70062

**Published:** 2025-09-09

**Authors:** Wu Pingyun, Wu Yuan, Wu Ruolan, Wan Xueting, Yang Qi, She Pengfei

**Affiliations:** ^1^ Department of Laboratory Medicine The Third Xiangya Hospital of Central South University Changsha China; ^2^ Department of Laboratory Medicine Shenzhen Hospital of Traditional Chinese Medicine Shenzhen China

**Keywords:** biofilm, closantel, drug repurposing, *Staphylococcus epidermidis*

## Abstract

*Staphylococcus epidermidis* is recognized as the major cause of implanted indwelling medical device‐related infections. The ability of *S. epidermidis* to form biofilms largely increases its resistance to conventional antibiotics, which is the major cause of treatment failure. Therefore, there is a pressing need to discover novel antimicrobials against *S. epidermidis* biofilms. In this study, Closantel (Clos), an antiparasitic drug, was repurposed to be effective against *S. epidermidis* planktonic cells with the minimal inhibitory concentration values of 0.25–0.5 μg/mL. Clos exhibited potent biofilm inhibition at ≥ 0.5 μg/mL and achieved effective eradication at ≥ 1 μg/mL. Notably, Clos induced lower resistance in *S. epidermidis* compared to Rifampicin. Mechanism study indicated that Clos exerted the bactericidal activity mainly through inducing bacterial cell membrane depolarization and further disruption. And the antibiofilm activity of Clos could be partially due to the inhibition of initial adhesion and extracellular polysaccharides production. In addition, CCK‐8 assay showed that Clos at 16 μg/mL had limited cytotoxicity in A2780, HaCaT and 293 T cells. In conclusion, this study demonstrates that Clos, a molecule targeting bacterial cell membranes, exhibits strong antimicrobial and antibiofilm effects in vitro against *S. epidermidis*. Although, side effects were reported in mammals, developing Clos derivatives could be still an effective therapeutic strategy to treat *S. epidermidis*‐related infections.

## Graphical abstract

This study innovatively explored the potential of repurposing the antiparasitic drug closantel, and revealed its previously unreported antibacterial and antibiofilm activity against *Staphylococcus epidermidis in vitro*.

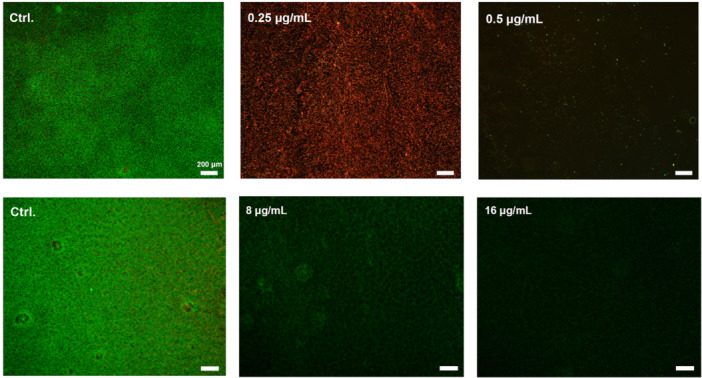

## 1 Introduction

*Staphylococcus epidermidis*, a commensal bacterium colonizing human and mammalian skin and mucosal surfaces (Severn and Horswill 2023), has emerged as the predominant pathogen responsible for implanted indwelling medical device‐related infections, including prosthetic valve endocarditis, joint infections, and even sepsis (Otto 2018; Schilcher and Horswill 2020). It was reported that followed to *Staphylococcus aureus* (Ramos‐Martínez et al. 2023), the incidence of coagulase‐negative *Staphylococcus* (CoNS)‐associated endocarditis caused by *S. epidermidis* was accounted for 79.4% (Haddad et al. 2023) with a high mortality of 24% (Otto 2009). With the abuse of antibiotics, *S. epidermidis* has developed wide resistance to conventional antibiotics. For example, Xu et al. (2020) reported that up to 76.5% of the 107 *S. epidermidis* clinical strains collected from Tianjin Hospital in China were resistant to methicillin. Moreover, methicillin‐resistant CoNS was also reported to be resistant to other classes of antibiotics, including vancomycin (VAN), penicillin, ciprofloxacin, erythromycin, or gentamicin (Otto 2009; Ibrahim and Abu El‐Wafa 2020).

Although *S. epidermidis* has fewer virulence factors than *S. aureus*, its strong ability to form biofilms could lead to refractory infections due to its substantial resistance to antibiotics and host immune defenses (Otto 2009; Zhu et al. 2021). Biofilm is a multicellular structured microbial community encapsulated by extracellular polymeric substances (EPS) consisting of polysaccharide, extracellular DNA (eDNA) and extracellular protein (eProtein) (Schilcher and Horswill 2020). Biofilms could resist immune cell penetration and limit antibiotic diffusion through extracellular barriers (Otto 2018; Rumbaugh and Sauer 2020; Gross et al. 2001), and also largely reduce the antimicrobial activity of drugs such as penicillin, aminoglycosides, and quinolones by dormant cells (Otto 2009), resulting in resistance being 10–1000 times that of the planktonic cells. Consequently, it is imperative for the development of novel antimicrobials that prominently target *S. epidermidis* biofilms.

Given the challenges in novel antimicrobial development, drug repurposing is an effective therapeutic strategy to prevent the emergence of drug resistance (Konreddy et al. 2019). Closantel (Clos) is a salicylanilide anthelmintic with broad‐spectrum antiparasitic activity against liver flukes, nematodes, blood‐sucking parasites and arthropods, with high plasma protein binding (> 99%) (Saleh et al. 2022; Jabbar et al. 2022; Michiels et al. 1987). Currently, several studies have reported its antibacterial potential. Clos exhibited antibacterial activities against Gram‐positive pathogens such as *S. aureus* (including MRSA USA300) and *Enterococcus faecalis*, and synergistic antibacterial activity against multidrug‐resistant Gram‐negative infections when combined with other drugs (Domalaon et al. 2019; Hlasta et al. 1998; Niu et al. 2017). In addition, Tang and Zhu (2024) further demonstrated that Clos effectively disrupts *S. aureus* membrane integrity and has therapeutic potential for *S. aureus* and its biofilm‐associated infections. However, the study of Clos for the treatment of *S. epidermidis* and its biofilm‐associated infection is currently unexplored.

In this study, we identified Clos as a membrane‐disrupting compound with potent bactericidal activity against *S. epidermidis*, and explored its underlying mechanisms. Given the significant antibacterial and antibiofilm activity of Clos against *S. epidermidis* with low resistance induction ability, developing safer derivatives based on its core scaffold is a highly promising antibacterial strategy for treating *S. epidermidis*‐related infections.

## 2 Materials and Methods

### 2.1 Strains, Culture Conditions, and Reagents

Di Qu from Fudan University in Shanghai, China generously provided the type strains of *S. epidermidis* RP62A (ATCC 35984, biofilm‐positive strain) and ATCC 12228 (biofilm‐negative strain). From the Third Xiangya Hospital of Central South University, clinical isolates of *S. epidermidis* were collected, and their identifications were confirmed using VITEK 2 Compact (bioMérieux, France) and matrix‐assisted laser desorption/ionisation time‐of‐flight mass spectrometry (BD, Germany). *S. epidermidis* was cultured in Tryptic Soy Broth (TSB) (Solarbio, Beijing, China) unless specifically mentioned. Dimethyl sulfoxide (DMSO) was used to dissolved in the following drugs in this study: Clos, VAN, and Rifampicin (RFP), all supplied by MedChem Express (New Jersey, USA).

### 2.2 Antimicrobial Susceptibility Test

The minimum inhibitory concentration (MIC) values of the antimicrobials were measured by a previously established standard method (CLSI 2024). Briefly, the log‐phased bacterial suspension was diluted to approximately 1.5 × 10^6^ CFU/mL using Mueller‐Hinton (MH) II broth (Solarbio, Beijing, China), and then incubated with serially diluted antimicrobials in equal volume for 16–8 h at 37°C. For the MIC, it was defined as the lowest concentration of the antimicrobials with no visible growth observed. Meanwhile, the minimum bactericidal concentration (MBC) was considered as the lowest antimicrobial concentration capable of reducing 99.9% of the initial colonies on sheep blood agar plate (Autobio, Zhengzhou, China).

### 2.3 Bactericidal Dynamics

Overnight cultured *S. epidermidis* was standardized to 1 × 10^6 ^ CFU/mL with fresh TSB in the absence or presence of the indicated concentrations (1/4 ~ 4×MIC) of Clos. The bacterial suspension with 180 rpm of shaking at 37°C was subsequently assayed for growth turbidity (OD_630nm_) at the specified time points (including 0, 2, 4, 6, 8, and 12 h). Meanwhile, ten microliters of the bacterial culture suspension were consecutively diluted in sterile saline, and dropped onto the sheep blood agar. The colony‐forming units (CFU) were then counted after incubation at 37°C for 24 h (Liu et al. 2021).

### 2.4 Spontaneous Resistance Assay

The spontaneous resistance frequency of *S. epidermidis* in the presence of RFP or Clos was determined by a single‐step resistance inducting. Briefly, overnight cultured *S. epidermidis* RP62A was adjusted to an OD_630nm_ of 0.5. Subsequently, 100 μL of the bacterial suspensions were then coated onto MH agar plate containing indicated concentrations of RFP or Clos. Following incubation for 48 h at 37°C, the mutant CFUs on each plate were counted (Zhou et al. 2020).

### 2.5 Biofilm Forming Capacity Assessment

The bacterial biofilm formation ability of *S. epidermidis* was assessed as previously reported (Campana et al. 2019) with minor adjustments. Firstly, overnight cultured bacteria were diluted in TSB to 1 × 10^6^ CFU/mL in a 96‐well plate. After cultivation at 37°C for 24 h, the attached biofilms were dyed with a 2% (wt/vol) crystal violet (CV) solution for 15 min and the excess dye was rinsed off with 1× phosphate buffer saline (PBS, pH = 7.4). The bound CV was solubilized in 95% ethanol for 30 min and then determined at 570 nm (A_570 nm_).

### 2.6 Biofilm Inhibition Assay

The overnight cultured *S. epidermidis* was adjusted to 1 × 10^6^ CFU/mL with TSB. An aliquot of the bacterial suspension with equal volume of specified concentrations of clos was added to a 96‐well cell culture plate or a 12‐well plate with a titanium (Ti) disc in each well. Following incubation at 37°C for 24 h, non‐adherent cells were cleared with PBS. And the remained biofilm was quantified using the CV staining method as mentioned above. Meanwhile, the biofilm was thoroughly scraped, mixed and resuspended in 200 µL of sterile saline. And the suspension was further consecutively diluted in saline, dropped on sheep blood agar and further incubated overnight for viable cell counting (Mary Mawumenyo Mamattah et al. 2023).

### 2.7 Biofilm Eradication Assay

Overnight cultured *S. epidermidis* was standardized to 1 × 10^6^ CFU/mL, and subsequently added 200 µL or 1 mL to a 96‐well or 12‐well (with a Ti disc in each well) cell culture plate, respectively. After cultivation at 37°C for 24 h, biofilms formed on the surface of the 96‐well plates or Ti discs were rinsed with saline and added with fresh TSB at specified concentrations of Clos. After cultured for further 24 h, the biofilms were determined by CV staining as well as CFU counting as described above (Jayathilaka et al. 2022).

### 2.8 Biofilm Observation by SYTO9/PI Staining

Briefly, 2 × 2 cm cover slides were placed on the bottom of a 6‐well cell culture plate. Overnight cultured *S. epidermidis* suspension (adjusted to 1 × 10^6^ CFU/mL) was added to each well with equal volume of TSB containing indicated concentrations of Clos for biofilm inhibition assay. And For biofilm eradication assay, the cultured bacterial suspension in each well for 24 h was further added with TSB containing indicated concentrations of Clos. After incubated at 37°C for 24 h, the biofilms on the coverslip surface were stained with SYTO9/PI probes (10 μM), and the live (green)/dead (red) cells in biofilms were then assessed by a fluorescence microscopy observation (Zeiss Vert A1, Germany) (Pengfei et al. 2022).

### 2.9 Initial Adhesion Assay

Log‐phased *S. epidermidis* was sub‐cultured in TSB containing 1% glucose, and then mixed with equal volume of Clos at the final concentrations of 1/4 to 4 × MIC. One percent of DMSO served as the control. To facilitate initial adherence, the bacterial suspension was incubated at 37°C for 4 h. And the non‐adherent cells were then removed with sterile saline and the OD_630nm_ of the wells was determined. Meanwhile, the adherent cells were thoroughly mixed in saline and serially diluted with sterile for CFU counting (Liu et al. 2019).

### 2.10 Determination of Biofilm Formation at the Air–Liquid Interface

Five milliliters of bacterial suspension (1 × 10^6^ CFU/mL) with equal volumes of indicated concentrations of Clos were added to glass tubes. After static cultivation for 24 h at 37°C, the tubes rinsed with PBS were air dried and subsequently dyed with 2% CV solution as previously mentioned. Finally, the biofilms at the air‐liquid interface were photographed (Cota et al. 2021).

### 2.11 eDNA and eProtein Quantification

Quantification of eDNA and eProtein was performed as the method previously mentioned(Shukla and Rao 2017), with slight modifications. Briefly, 2 mL of overnight cultured *S. epidermidis* suspension was cultured with TSB in a 6‐well plate for 24 h. The pre‐formed biofilm was gently rinsed twice with PBS for removal of planktonic cells. After treated with clos for 4 h, the biofilm was dispersed by a Sonic Vibra Cell VCX processor. After centrifuged at 8000 rpm and 4°C for 30 min, the supernatant was then combined with twice the volume of 95% ethanol and kept at 4°C overnight. Subsequently, the mixture was centrifugated at 10,000 rpm for 10 min and the resulting precipitate was dried at 60°C and further dissolved in 100 µL of PBS. Finally, quantification of eDNA and eProtein were measured with NanoDrop 2000 spectrophotometer (Thermo Fisher Scientific, Waltham, MA) at 260 nm and 280 nm, respectively.

### 2.12 Congo Red Agar Assay

The Congo red agar was prepared with basic medium components (including TSB, sucrose and agar) and Congo red dye autoclaved separately at 121°C for 15 min, which were mixed together once the agar had cooled to 55°C. Overnight cultured *S. epidermidis* was cultured onto Congo red agar medium. After incubation for 1–2 days, the color of the colonies was observed. It is considered the biofilm forming positive when the colony is red, but negative when it is black (Cho et al. 2022).

### 2.13 Hydrophobicity Assay

The hydrophobicity assay was carried out as previously mentioned by Song et al (Song et al. 2019). Overnight cultured *S. epidermidis* was treated with or without 1/2 × MIC of Clos for 3 h. After centrifugated at 3000 rpm for 10 min, the precipitate was resuspended with PBS to an OD_630nm_ of 0.5 ± 0.05 (OD _initial_). Two milliliters of the bacterial suspension were thoroughly vortexed with 0.5 mL of xylene for 2 min and then incubated for 15 min. The OD value of the lower aqueous phase was measured at 630 nm (OD _treatment_). The hydrophobicity was calculated as follows:

$$\text{Hydrophobicity}(\%)=\;(1\;–\;\frac{\;\text{treatment}}{\;\text{initial}})\times \;100\%.$$

### 2.14 Cell Transmembrane Potential Determination by DiSC3(5) Staining

The impact of Clos on the depolarization of *S. epidermidis* cell membrane was assessed by the fluorescent dye DiSC3(5). Briefly, log‐phased RP62A was resuspended in 5 mM HEPES buffer to an OD_630nm_ of 0.05, and further combined with the mixed solution (including 2 μM Disc3 (5) (AAT Bioquest, USA), 100 mM KCl, and 5 mM glucose) for 1 h in the dark. Clos was added to the bacterial suspension to the final concentration of 1/8 ~ 1 × MIC, respectively. The fluorescence intensity was recorded every 30 s for 5 min at the excitation and emission wavelengths of 622 and 670 nm, respectively. As controls, DMSO were served as a negative control and melittin (1 × MIC) as a positive control (Dong et al. 2019).

### 2.15 Bacterial Cell Membrane Permeability Detection by PI Dyeing

The bacterial suspension with an OD_630_ of 0.05 were prepared as described above, then 10 μM of PI was added and incubated for 30 min in the dark. Finally, the fluorescence was measured every 5 min for 30 min at the excitation and emission wavelengths of 535 and 617 nm (Ma et al. 2019).

### 2.16 Detection of Cytotoxicity by the CCK‐8 Kit

The cytotoxicity of Clos against the cell lines of A2780 (Human ovarian cancer cell line), HaCaT (Human immortalize epidermal cell line) and 293 T (Human renal epithelial cell line), was determined by the CCK‐8 assay. For each type of cell line, log‐growth phase culture was established in DMEM medium supplemented with 1% antibiotics and 10% fetal bovine serum (Kaiji Biotechnology Development Co, Nanjing, China) at 37°C. Cells were plated into 96‐well plates at a density of 2 × 10^3^ cells per well and exposed to the Clos‐containing medium for 24 h. The cell suspension with the addition of 10 μL of CCK‐8 solution (Dojindo, Japan) was cultured for 1.5 h at 37°C, and then measured at OD_450nm_ (Luo et al. 2021).

### 2.17 Statistical Analysis

All experiments were performed independently in triplicate. All data were analyzed by GraphPad Prism 8.0 software and presented as mean ± standard deviation (SD). Student's *t*‐test was applied to compare the statistical difference between two groups, while one‐way ANOVA was applied to detect the difference more than two groups. *p* < 0.05 was considered statistically significant.

## 3 Results

### 3.1 Effective Antimicrobial Activity of Clos Against *S. epidermidis* Planktonic Cells

**Figure 1:**
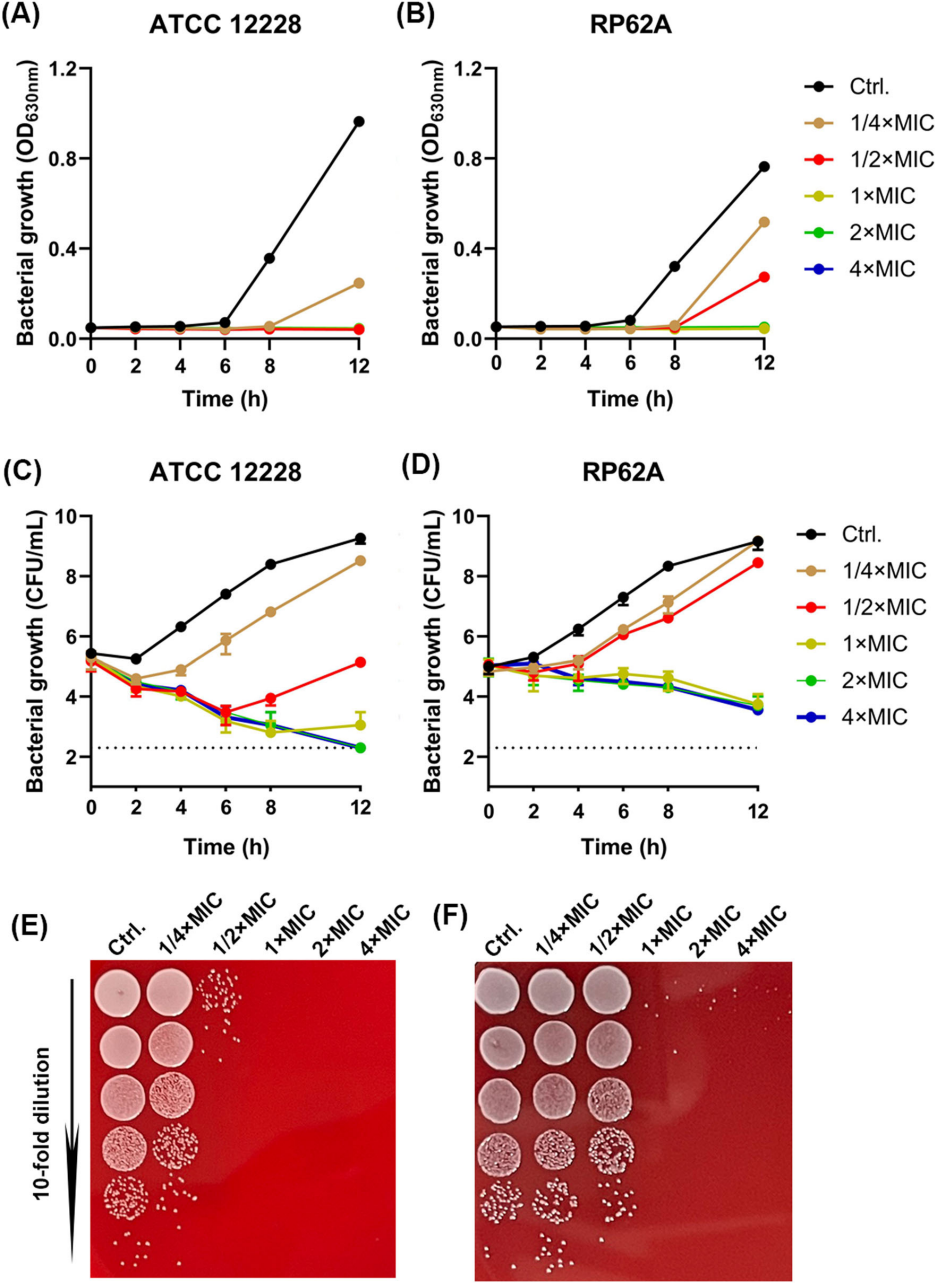
Bactericidal activity of Clos against *S. epidermidis*. Time‐inhibition curves of Clos against *S. epidermidis* ATCC 43300 (A) and RP62A (B), respectively. Bactericidal kinetics of Clos against ATCC 12228 (C) and RP62A (D), respectively. Representative images of CFU counting at the time point of 12 h for ATCC 12228 (E) and RP62A (F), respectively.

The antimicrobial susceptibility of Clos against *S. epidermidis* type strains and clinical isolates was evaluated by micro‐dilution method. As presented in Table 1, Clos showed significant bactericidal activity against *S. epidermidis*, achieving the MIC and MBC values in the range of 0.25–0.5 and 0.25–8 µg/mL, respectively, while VAN showed the MIC and MBC values of 2–8 µg/mL for all tested strains. Accordingly, the bactericidal kinetics of Clos were evaluated. As shown in Figure 1A,B, sub‐MIC of Clos (1/4 × MIC) could show significant growth inhibition activity against *S. epidermidis* strains within 12 h. And the growth was completely inhibited, in a concentration‐dependent manner, by 1/2 × MIC and 1/4 × MIC of Clos for ATCC 12228 (Figure 1A) and RP62A (Figure 1B), respectively. Sub‐MIC (1/2 × MIC) of Clos could decrease the viable cell counts of ATCC 12228 by 4.10 log_10_ CFU/mL after 12 h treatment (Figure 1C). Meanwhile, 1 × MIC of Clos also exhibited significant bactericidal activity against RP62A with a bacterial reduction by 6 log_10_ CFU/mL (Figure 1D). Similarly, representative images showed no colony growth of ATCC 12228 (Figure 1E) and RP62A (Figure 1F) on sheep blood agar plates in the presence of 1/2 × MIC and 1 × MIC of Clos, respectively. Then, the resistance induction ability and cytotoxicity of Clos were assessed. As shown in Table 2, the Clos‐treated group showed no growth of mutant colonies at 8×MIC, whereas the RFP‐treated group showed a significant number of mutant colonies at the same concentration. In addition, the number of mutant colonies in the Clos‐treated group at 16 × MIC was reduced compared to that in the RFP‐treated group, suggesting that Clos inhibited drug‐resistant mutations in *S. epidermidis* compared to RFP. In addition, no significant cytotoxicity against the cell lines of A2780, HaCaT and 293 T was found when treated with 16 μg/mL of Clos (Supporting Information S1: Figure S1).

**Table 1:** Antimicrobial susceptibility of molecules against *S. epidermidis*.

Strains	Closantel		VAN	
	MIC (μg/mL)	MBC (μg/mL)	MIC (μg/mL)	MBC (μg/mL)
Type strain				
RP62A	0.25	1	2	2
ATCC 12228	0.5	8	2	2
Clinical isolates				
S1	0.25	1	2	2
S2	0.25	0.5	2	2
S3	0.25	2	2	2
S4	0.25	0.25	2	2
S5	0.25	2	2	4
S6	0.5	4	2	2
S7	0.25	2	2	4
S8	0.25	8	2	2
S9	0.5	2	4	8
S10	0.5	2	2	2
S11	0.5	2	2	2
S107	0.25	1	2	2
S108	0.5	4	2	2
S109	0.5	8	2	2

**Table 2:** Resistance occurrence of closantel and RFP.

Antimicrobials	Closantel						RFP					
	8 × MIC			16 × MIC			8 × MIC			16 × MIC		
Initial CFUs (×10^6^ CFU/mL)	260	290	270	260	290	270	260	290	270	260	290	270
Mutant CFUs (CFU/mL)	0	0	0	0	0	10	50	70	80	0	0	150

### 3.2 Strong Antibiofilm Activity of Clos Against *S. epidermidis*

**Figure 2:**
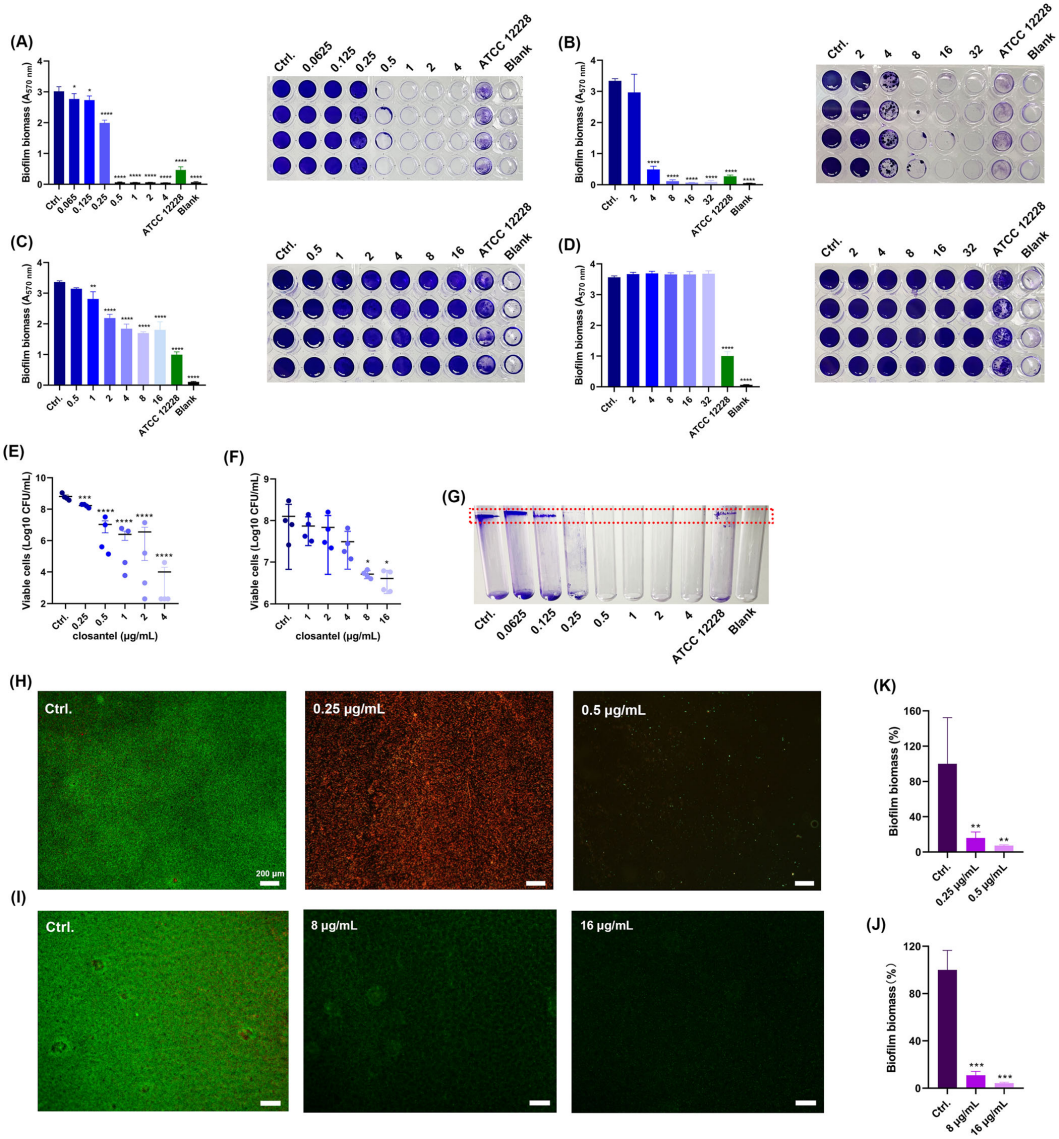
Antibiofilm effects of Clos or VAN against *S. epidermidis* type strain RP62A. (A) Biofilm inhibition activity of Clos against RP62A. (B) Biofilm inhibition activity of VAN against RP62A. (C) Biofilm eradication activity of Clos against RP62A. (D) Biofilm eradication activity of VAN against RP62A. Viable cells counting for biofilm inhibition (E) and eradication (F), respectively, by Clos. (G) The biofilm formation at the air–liquid interface of *S. epidermidis* in the presence or absence of Clos. (H) Biofilm inhibition observed by SYTO9/PI staining. (I) Biofilm eradication observed by SYTO9/PI staining. Scale: 200 µm. Quantification of fluorescence intensity for biofilm inhibition (K) and eradication (J), respectively, against RP62A. *p < 0.05; **p < 0.01; ***p < 0.001; ****p < 0.0001.

Firstly, we determined the antibiofilm activity of Clos against *S. epidermidis* by CV staining. Clos at 0.5 μg/mL or higher significantly suppressed the RP62 A biofilm formation (Figure 2A), whereas only moderate biofilm inhibitory effect was observed by 4 μg/mL of VAN (Figure 2B). Furthermore, the pre‐formed biofilm of RP62A exposed to 1 μg/mL Clos was eliminated in a dose‐dependently manner (Figure 2C). However, no biofilm eradicating effect was found by VAN at the concentration even up to 32 μg/mL (Figure 2D). Meanwhile, the antibiofilm effects were also detected by CFU counting assay. For biofilm inhibition, the viable cells showed a significant reduction after treated with 0.25 µg/mL of Clos, and 0.5 µg/mL of Clos could inhibit the biofilm formation by 2.46 Log_10_ CFU/mL in comparison to the untreated group (Figure 2E). Similarly, the pre‐formed biofilm biomass exposed to 8 µg/mL of Clos was significantly reduced by 1.25 Log_10_ CFU/mL (Figure 2F). In addition, 1/2 × MIC (0.125 µg/mL) of Clos could significantly inhibit the biofilm formation by RP62A at the air–liquid interface (Figure 2G). By LCSM observation, a significantly reduction in the ratio of live/dead cells was found when exposed to 0.25 µg/mL of Clos compared to the control, and almost no bacterial cell was observed after treatment with 0.5 µg/mL of Clos (Figure 2H,K). And the obvious antibiofilm effects of Clos at the concentration of 8–16 μg/mL against established biofilms were also observed by the LCSM (Figure 2I,J).

**Figure 3:**
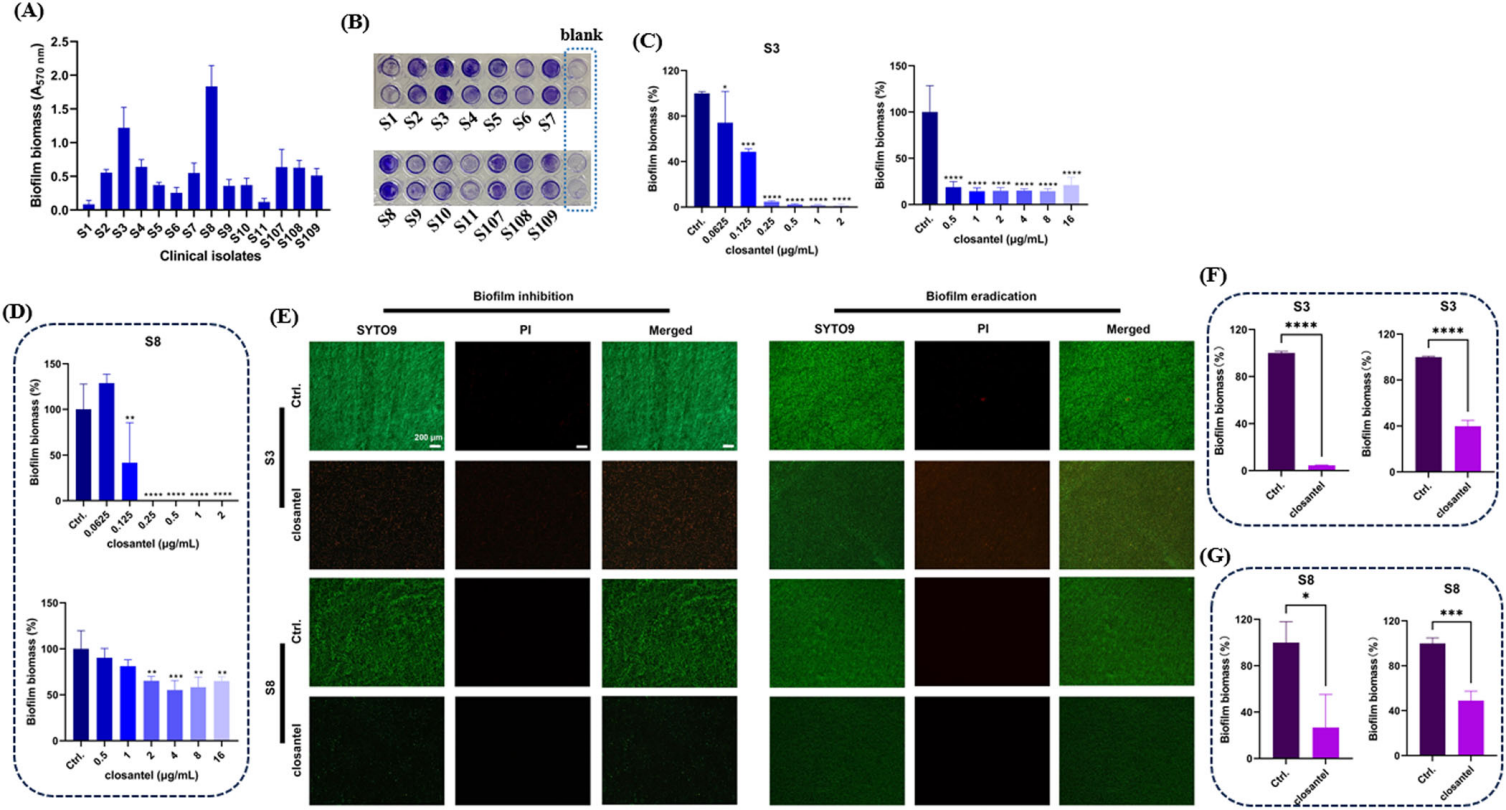
Antibiofilm effects of Clos against *S. epidermidis* clinical strains. (A) The biofilm forming capacity of 14 clinical strains of *S. epidermidis*. (B) The biofilm formation of clinical strains observed by CV staining. (C) Quantification of the biofilm inhibition and eradication by Clos against S3 using CV staining. (D) Quantification of the biofilm inhibition and eradication by Clos against S8 using CV staining. (E) The biofilm inhibition (Clos: 0.25 µg/mL) and eradication (Clos: 2 µg/mL) of Clos against S3 and S8 observed by SYTO9/PI staining. Scale: 200 µm. Statistical analysis of the biofilm fluorescence intensity of S3(F) and S8(G), respectively. *p < 0.05; **p < 0.01; ***p < 0.001; ****p < 0.0001.

The antibiofilm activities of Clos against *S. epidermidis* clinical strains were also determined. Firstly, the biofilm forming capacity of 14 *S. epidermidis* clinical strains was assessed by CV staining. As shown in Figure 3A,B, the strains of S3 and S8 exhibited the strongest biofilm forming capacity among the tested strains, which were selected for further studies. By CV staining, Clos was found to significantly inhibit the biofilm formation and eradicate the pre‐formed biofilms of S3 at the concentration of 0.0625 and 0.5 µg/mL, respectively (Figure 3C). Similarly, the significant antibiofilm effects of Clos at the concentration of 0.125 and 2 µg/mL against S8 were also observed for biofilm inhibition and eradication, respectively (Figure 3D). LCSM showed that the total biofilm biomass of S3 and S8 was obviously reduced and the proportion of dead cells was significantly increased after treated with Clos (Figure 3E), which indicated the strong biofilm inhibitory and eradicating activity by Clos. In consistent, by quantifying of the fluorescence intensity, the proportion of viable cells (green) by S3 exhibited 4.43% and 39.85% reduction for biofilm inhibition and eradication assays, respectively (Figure 3F). Similarly, 26.72% and 48.91% reduction by S8 were observed for the biofilm inhibition and eradication, respectively (Figure 3G).

### 3.3 Antibiofilm Effects of Clos Against *S. epidermidis* on Ti‐Discs

**Figure 4:**
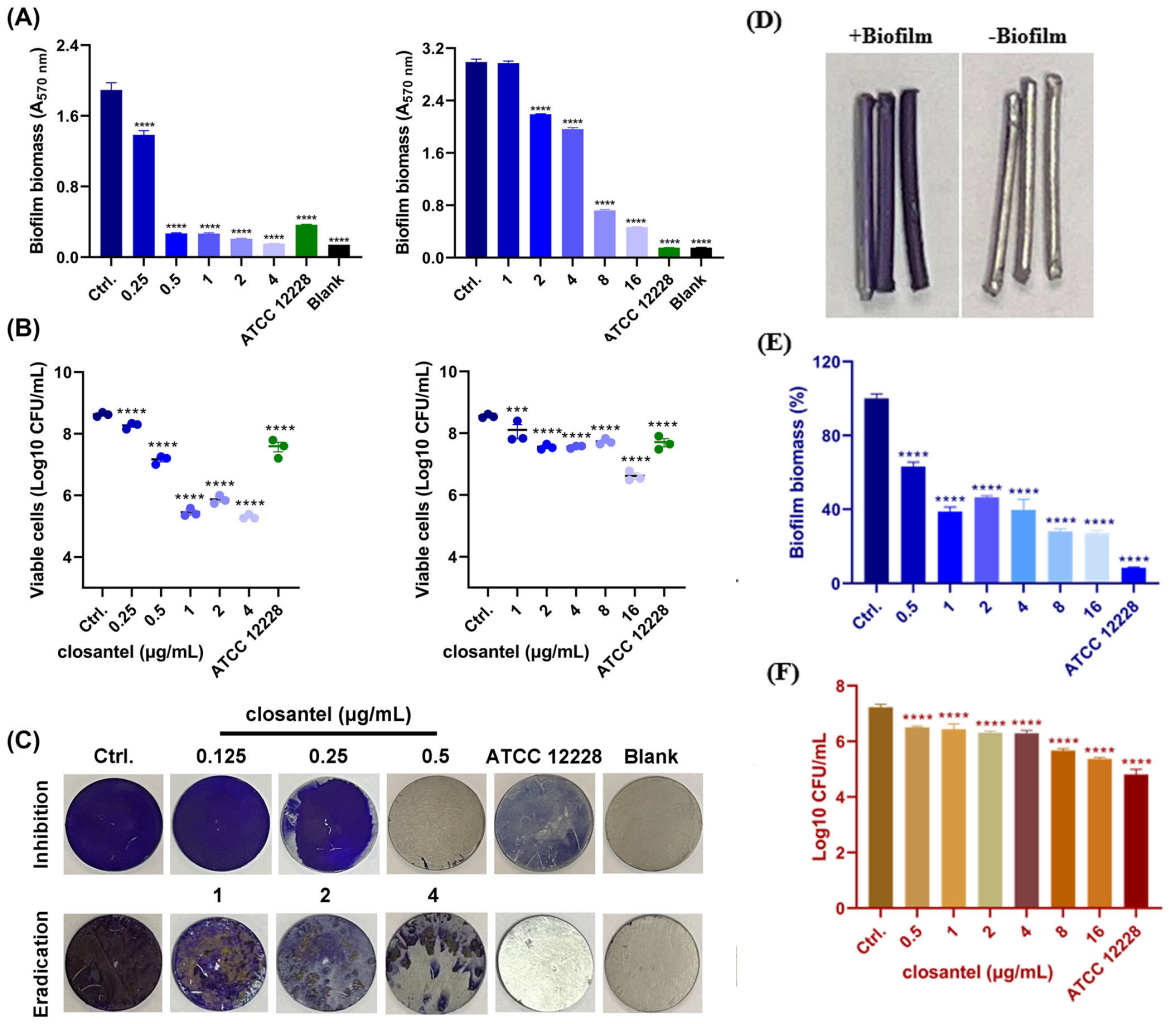
Antibiofilm effect of Clos against *S. epidermidis* on Ti. (A) The biofilm inhibition and eradication activity of Clos against RP62A on Ti discs determined by CV staining. (B) The viable cells counting in the biofilm inhibition and eradication assays on Ti discs after treated with Clos. (C) Representative images of biofilm formation on Ti discs observed by CV staining. (D) Representative images of the biofilm formed on Ti needles determined CV staining. (E–F) Antibiofilm effects of Clos against *S. epidermidis* on Ti needles quantified by CV staining (E) and CFU counting (F), respectively. *p < 0.05; **p < 0.01; ***p < 0.001; ****p < 0.0001.

Ti is a commonly used medical material for medical prosthesis implantation (Chouirfa et al. 2019). In this study, the prominent antibiofilm effects of Clos against *S. epidermidis* on Ti‐discs were detected. The CV staining on Ti‐discs showed that the RP62A biofilm exposed to 0.25 and 2 µg/mL of Clos was significantly inhibited and further eradicated, respectively (Figure 4A,C). In consistent, by CFU counting, Clos was found to significantly reduced the viable cells in the biofilm inhibition and eradication assays (Figure 4B). However, the decreased viable cell counts in the pre‐formed biofilms was not obviously correlated with the quantitative analysis by CV staining in Figure 4A (right panel), which was probably due to the more significant extracellular matrix reduction than directly bacterial cells killing by Clos treatment. Similarly, we successfully cultured the biofilms on Ti‐needles (Figure 4D), and the biofilm eradication activity by Clos was also observed by CV staining (Figure 4E) and CFU counting (Figure 4F), respectively.

### 3.4 Antimicrobial and Antibiofilm Mechanisms of Clos

Given the rapid bactericidal activity of Clos against planktonic cells of *S. epidermidis*, the impact of Clos on the cytoplasmic membrane was investigated. Firstly, the depolarization of bacterial cell membrane was determined by using DiSC3(5) probe. As shown in Supporting Information S1: Figure S2A, the intensity of fluorescence was dose‐dependently decreased when exposed to 1/8–1 × MIC of Clos. As we expected, the damaged cell membrane was further validated by using PI probe (Supporting Information S1: Figure S2B).

**Figure 5:**
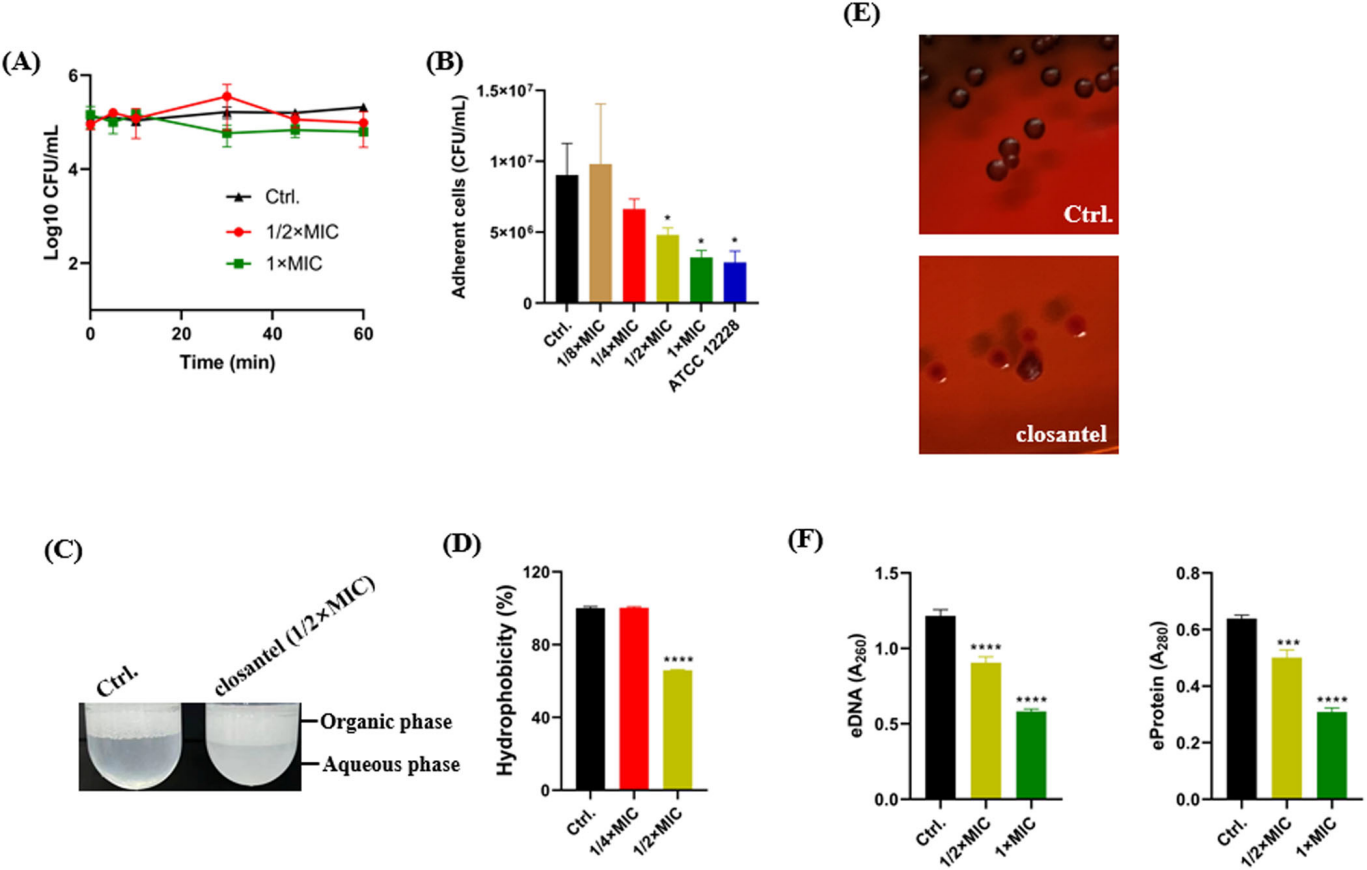
Preliminary exploration of the antibiofilm mechanisms by Clos. (A) Bactericidal activity of Clos against *S. epidermidis* within 1 h. (B) The bacterial cells counting of the initial adhesion after the treatment of Clos for 30 min. (C) Representative images of the hydrophobic ability of *S. epidermidis* after treated with Clos for 2.5 h. (D) Quantification of hydrophobic activity by RP62A in the presence or absence of Clos. (E) Representative images of the EPS secretion by *S. epidermidis* after treated or untreated with Clos. (F) Quantification analysis of the eDNA and eProtein after the treatment of Clos for 4–5 h. *p < 0.05; ***p < 0.001; ****p < 0.0001.

Next, the potential antibiofilm‐related mechanisms were also explored. Although, no difference was detected between the Clos (1 × MIC) treated group and the control group by CFU counting within 1 h (Figure 5A), a significant inhibitory effect by Clos against the initial adhesion was observed (Figure 5B). As we expected, the hydrophobic ability, associated with the initial adhesion capacity, was also decreased after treated with 1/2 × MIC of Clos (Figure 5C,D). In addition, as assessed by Congo red agar, the EPS production was obviously reduced after the treatment. It was shown that RP62A formed black colonies in the untreated group after 48 h incubation, while the Clos‐treated group observed with red colonies (Figure 5E). Furthermore, Clos could also significantly reduce the production of eDNA and eProtein by RP62A (Figure 5F).

## 4 Discussion

*S. epidermidis* has become the most important pathogen in biofilm‐associated infections associated with indwelling medical devices (Lister and Horswill 2014), with antibiotic resistance making the treatment challenging. Due to the long development and high cost of *de novo* drug (Konreddy et al. 2019), this study innovatively explored the repurposing potential of the antiparasitic drug Clos.

In this study, we found that Clos exhibited significant in vitro bactericidal and antibiofilm effects against *S. epidermidis*. Mechanism study indicated that Clos induced depolarization of the cell membrane, and the PI probe revealed that 1 × MIC Clos significantly enhanced *S. epidermidis* membrane permeability and finally achieved membrane disruption. These suggests that the cell membrane is a target for Clos to kill planktonic bacteria rapidly. Currently, most membrane‐targeted antimicrobials caused rapid bacterial death by enhancing the cell membranes permeability through electrostatic interactions between surface cations and negatively charged cell membranes, resulting in disruption of the bacterial proton motive force (Yeaman and Yount 2003) and further lead to the leakage of bacterial contents (Ahmed and Hammami 2019). Additionally, the electrostatic affinity of these antimicrobials to bacterial membranes often exhibits low selectivity for mammalian amphiphilic cell membranes, suggesting their potential for the clinical treatment of bacterial infections (Ganesan et al. 2023). Recently, researchers have reported many antimicrobials exerted their antibacterial activities through interactions with cell membrane (Li et al. 2021). For example, Klubthawee et al. (2023) found that CM‐10K14K could enhance the depolarization and permeability of *S. epidermidis* membranes within 5 min, and the cell membrane damage with dispersed intracellular contents was observed by electron microscopes. Similarly, the antimicrobial compound Balsacone C was revealed to induce cell membranes damage of methicillin‐resistant *S. aureus* leading to the release of intracellular DNA and proteins, and further achieve bactericidal activity (Côté et al. 2019).

It is reported that the antimicrobial molecules targeting bacterial cell membranes not only exhibit wide antimicrobial activity against negatively charged Gram‐positive bacteria, but also exert extremely low probability of bacterial resistance induction (Kim et al. 2020). In our study, Clos induced lower resistance in *S. epidermidis* compared to RFP. And Tang and Zhu (2024) reported that Clos hardly induced resistance of *S. aureus* with the MIC of Clos remaining unchanged in the 25‐day resistance induction assay. Therefore, compounds targeting membrane disruption, such as Clos, have a significant low probability of resistance induction in combatting multidrug‐resistant bacterial infections.

It is well known that biofilm formation on indwelling medical devices is considered to be the most important virulence factor of *S. epidermidis*‐associated. However, conventional antibiotics exert their antimicrobial effects mainly by targeting planktonic cells rather than their biofilm counterpart (Otto 2009). In previous studies, cinnamaldehyde (Albano et al. 2019), β‐Lapachone (Mir et al. 2023), and the combination of β‐Lactams and Branched Polyethylenimine (Lam et al. 2019) have been preliminarily explored for their potential to combat *S. epidermidis* biofilms. However, in this study, Clos showed significant inhibition of *S. epidermidis* biofilm formation and eradication of its pre‐existing biofilms at the concentrations of 0.5 and 1 µg/mL, respectively. In addition, Ti and its alloys are currently the most widely utilized metallic materials in the clinic settings for prosthetic bone implants, and the implant‐related infections remain a major cause of treatment failure due to the biofilm formation on Ti surfaces (Chouirfa et al. 2019). As we expected, Clos was effective in the biofilm inhibition and further eradication of the pre‐formed biofilms on the Ti surfaces.

Previous studies have reported that the biofilm formation process including three main stages: (1) initial adhesion to biotic or abiotic surfaces; (2) formation of biofilm structures by cell aggregation, EPS secretion and bacterial proliferation; and (3) mature biofilm dispersion and seeded to new colonization sites (Otto 2009). As previously reported, *S. epidermidis* relied mainly on hydrophobic interaction for its initial adhesion to the medical equipment surface (Vuong and Otto 2002). Initial adhesion is the first step of biofilm formation, initiating the entire biofilm process and laying the foundation for the subsequent EPS secretion (Otto 2009). It has been reported that Quebrachitol significantly inhibited *S. epidermidis* biofilm formation in a concentration‐dependent manner by inhibiting the bacterial initial adhesion and decreasing the auto‐aggregation ability (Karuppiah and Thirunanasambandham 2020). Similarly, in our study, we found that sub‐MIC of Clos (1/2 × MIC) inhibited bacterial initial adhesion mainly through the disruption of hydrophobicity, which further effectively inhibited the early biofilm formation, blocked EPS secretion and prevented the biofilm maturation.

After the biofilm maturation, *S. epidermidis* exhibits strong resistance to conventional antibiotics and host defense due to their complex structure enveloped by EPS, making the infections extremely difficult to be cured (Lister and Horswill 2014). Therefore, it is essential to evaluate the effectiveness of antimicrobial compounds against the pre‐formed biofilms. In our study, the antimicrobial effect of Clos against pre‐formed biofilms was observed visually by LCSM, and the decreased secretion of EPS, eDNA, and eProtein was also qualitatively measured by Congo red agar and spectrophotometer, respectively. Although, the structural integrity of the mature biofilms is the most important factor for its resistance to antibiotics, Clos was demonstrated to be effective against *S. epidermidis* pre‐formed biofilms by extracellular matrix disruption. This indicates that the core scaffold of Clos may have high potential in combatting chronic and persistent infections caused by *S. epidermidis* biofilm.

Clos exhibits good pharmacokinetic properties in ruminants and the elimination half‐life in sheep and cattle was up to 2–3 weeks after oral or intramuscular administration (Michiels et al. 1987). In addition, Tang et al. reported that Clos, at the concentration of 64 μg/mL, showed limited cytotoxicity to hepatocytes (HepG2/LO2) in vitro. In consistent, in our study, Clos was exhibited minimal cytotoxicity against the cell lines of A2780, HaCaT and 293 T at the range of MICs. Despite the effective antimicrobial activity and low cytotoxicity of Clos, several studies have suggested that Clos may induce potential ocular toxicity, such as visual impairment and retinopathy (Van der Lugt and Venter 2007; O Leary et al. 2023; Ghods et al. 2021). And FAO/WHO propose to establish a maximum daily intake threshold of ≤ 0.03 mg/kg for Clos (WHO 1990). Furthermore, the European Medicines Agency explicitly prohibits the use of veterinary Clos in any preparations intended for human application. However, developing safe derivatives with reduced side effects while enhanced antimicrobial activity based on its scaffolds is still an attractive antibacterial strategy. For example, structural optimization of chloramphenicol yielded N‐phenyl‐2,2‐dichloroacetamide, which achieve controlled drug release and reduced hematological toxicity (Al Khatib et al. 2025).

## 5 Conclusions

Clos exerts significant antimicrobial and antibiofilm effects against *S. epidermidis in vitro* by increasing cell membrane permeability as well as interacting with initial adhesion and EPS production. Given the side effects of Clos in mammals, these results suggest that developing Clos derivatives targeting cell membranes according to its core scaffold have promising prospects for treating infections caused by *S. epidermidis* and its biofilm.

## Author Contributions

**Wu Pingyun:** conceptualization (lead), formal analysis (lead), investigation (lead), methodology (lead), visualization (lead), writing – original draft preparation (lead), writing – review and editing (equal). **Wu Yuan:** conceptualization (lead), methodology (lead); visualization (supporting), supervision (supporting), writing – original draft preparation (lead), writing – review and editing (equal). **Wu Ruolan:** formal analysis (supporting), methodology (supporting), writing – review and editing. **Wan Xueting:** formal analysis (supporting), investigation (supporting), writing – review and editing (equal). **Yang Qi:** formal analysis (supporting), visualization (supporting), writing – review and editing (equal). **She Pengfei:** conceptualization (supporting), formal analysis (supporting), funding acquisition (lead), methodology (supporting), project administration (supporting), supervision (lead), writing – original draft preparation (supporting), writing – review and editing (equal).

## Ethics Statement

The authors have nothing to report.

## Conflicts of Interest

The authors declare no conflicts of interest.

## Supporting information

20250425 Supplementary Material.

## Data Availability

All data generated or analyzed during this study are included in this published article.
