# Supplementary material for: Antibacterial and Antibiofilm Activity of Closantel Against Staphylococcus epidermidis
Source: Microbiologyopen. 2025 Sep 9;14(5):e70062. doi: 10.1002/mbo3.70062 (PMC12420361; doi:10.1002/mbo3.70062)
Supplement: Supplementary file 1 — 20250425 Supplementary Material. [file MBO3-14-e70062-s001.docx]

**Supplementary Material**

**
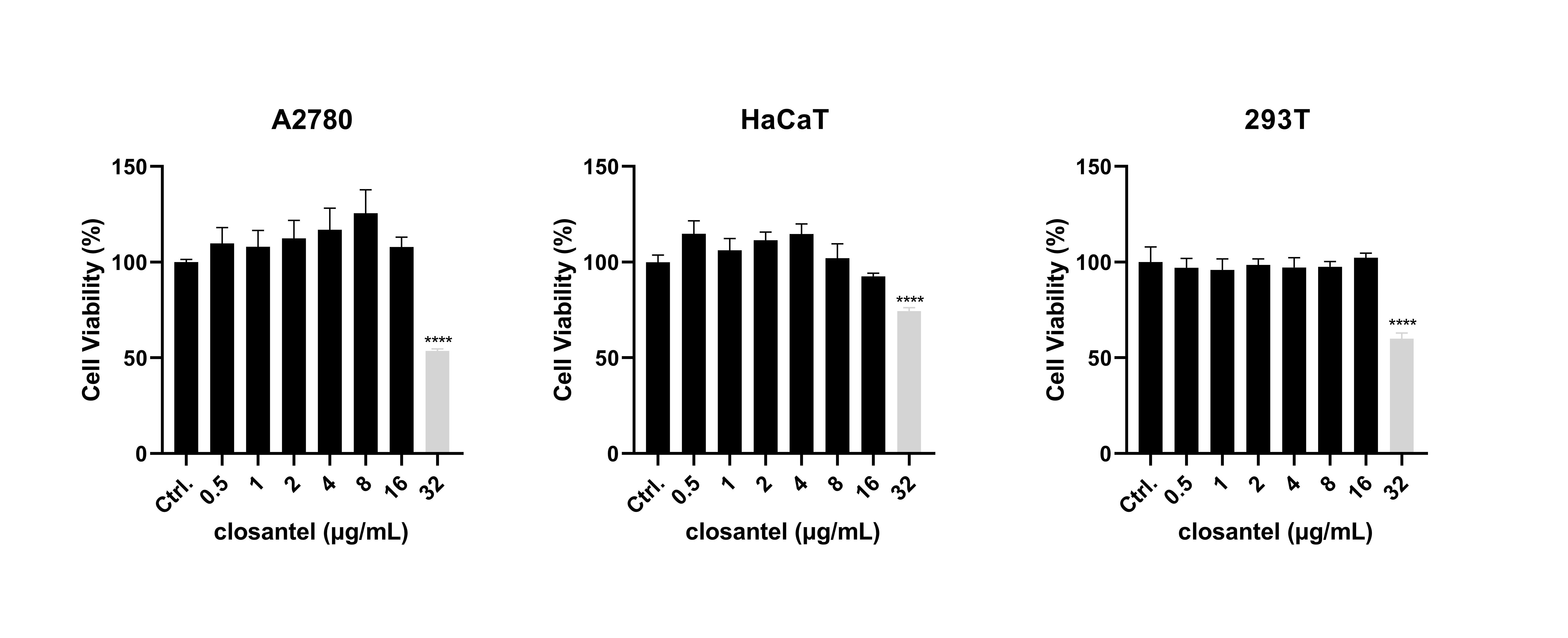
**

**Figure S1** *In vitro* cytotoxicity assessment of Clos by CCK-8 kit. The cell lines of A2780, HaCaT and 293T were treated with Clos for 24h. ****: *p* < 0.0001.


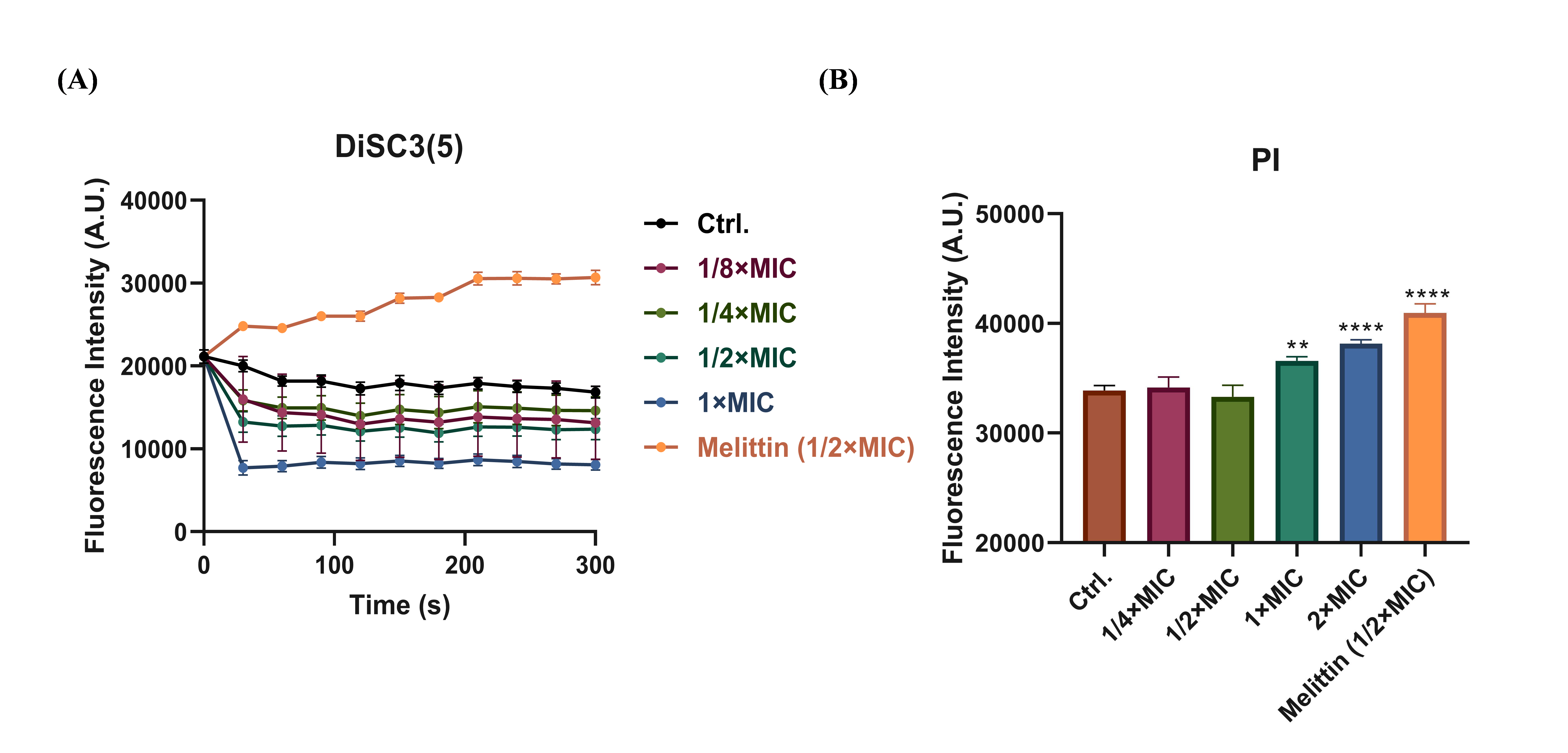


**Figure S2** Bacterial cell membrane disruption activity by Clos. (A) Bacterial cell membrane potential determination by DiSC3(5) probe. (B) The cell membrane permeabilization determination by PI probe**.** **: *p* < 0.01; ****: *p* < 0.0001.
